# Kaempferol: A Review of Current Evidence of Its Antiviral Potential

**DOI:** 10.3390/ijms242216299

**Published:** 2023-11-14

**Authors:** Argyrios Periferakis, Aristodemos-Theodoros Periferakis, Lamprini Troumpata, Konstantinos Periferakis, Andreea-Elena Scheau, Ilinca Savulescu-Fiedler, Ana Caruntu, Ioana Anca Badarau, Constantin Caruntu, Cristian Scheau

**Affiliations:** 1 Department of Physiology, The “Carol Davila” University of Medicine and Pharmacy, 050474 Bucharest, Romania; 2 Akadimia of Ancient Greek and Traditional Chinese Medicine, 16675 Athens, Greece; 3 Elkyda, Research & Education Centre of Charismatheia, 17675 Athens, Greece; 4 Pan-Hellenic Organization of Educational Programs (P.O.E.P), 17236 Athens, Greece; 5 Department of Radiology and Medical Imaging, Fundeni Clinical Institute, 022328 Bucharest, Romania; 6 Department of Internal Medicine, The “Carol Davila” University of Medicine and Pharmacy, 050474 Bucharest, Romania; 7 Department of Internal Medicine and Cardiology, Coltea Clinical Hospital, 030167 Bucharest, Romania; 8 Department of Oral and Maxillofacial Surgery, “Carol Davila” Central Military Emergency Hospital, 010825 Bucharest, Romania; 9 Department of Oral and Maxillofacial Surgery, Faculty of Dental Medicine, “Titu Maiorescu” University, 031593 Bucharest, Romania; 10 Department of Dermatology, “Prof. N.C. Paulescu” National Institute of Diabetes, Nutrition and Metabolic Diseases, 011233 Bucharest, Romania; 11 Department of Radiology and Medical Imaging, “Foisor” Clinical Hospital of Orthopaedics, Traumatology and Osteoarticular TB, 021382 Bucharest, Romania

**Keywords:** kaempferol, kaempferol derivatives, antiviral actions, physiopathology, traditional medicine, ethnobotany

## Abstract

Kaempferol and its derivatives are flavonoids found in various plants, and a considerable number of these have been used in various medical applications worldwide. Kaempferol and its compounds have well-known antioxidant, anti-inflammatory and antimicrobial properties among other health benefits. However, the antiviral properties of kaempferol are notable, and there is a significant number of experimental studies on this topic. Kaempferol compounds were effective against DNA viruses such as hepatitis B virus, viruses of the alphaherpesvirinae family, African swine fever virus, and pseudorabies virus; they were also effective against RNA viruses, namely feline SARS coronavirus, dengue fever virus, Japanese encephalitis virus, influenza virus, enterovirus 71, poliovirus, respiratory syncytial virus, human immunodeficiency virus, calicivirus, and chikungunya virus. On the other hand, no effectiveness against murine norovirus and hepatitis A virus could be determined. The antiviral action mechanisms of kaempferol compounds are various, such as the inhibition of viral polymerases and of viral attachment and entry into host cells. Future research should be focused on further elucidating the antiviral properties of kaempferol compounds from different plants and assessing their potential use to complement the action of antiviral drugs.

## 1. Introduction

Kaempferol (3,5,7-trihydroxy-2-(4-hydroxyphenyl)-4H-chromen-4-one) carries the name of Engelbert Kaempfer, a German doctor, naturalist, and historian [1]. It is a flavonoid which was initially discovered in *Camelia sinensis* [2] and is found in a great variety of plants [3]. The chemical compound has anticarcinogenic [4,5,6], anti-inflammatory [7], anti-adipogenic [8], hepatoprotective [9,10], cytoprotectivre/antioxidant [11], anti-venom [12], and antimicrobial effects [3]. Other polyphenols and phytochemicals share these types of effects (e.g., [13,14,15,16,17,18,19,20,21,22,23,24,25,26,27]), and plant phenolic extracts are under investigation for their medicinal uses [28,29,30,31,32,33].

The risk of viral diseases is ever increasing with the frequent emergence of new viral pathogens; meanwhile, effective antiviral treatments are in many cases non-existent or suboptimal [34]. The current state of antiviral treatment options reflects the need of novel solutions for combatting viral infections [35]. During the last few decades, the focus toward phytochemistry and phytomedicine has increased, as can be seen by the multitude of relevant scientific literature [36,37,38,39,40,41,42,43,44,45,46,47,48,49,50,51,52,53,54,55,56,57,58,59].

**Figure 1:**
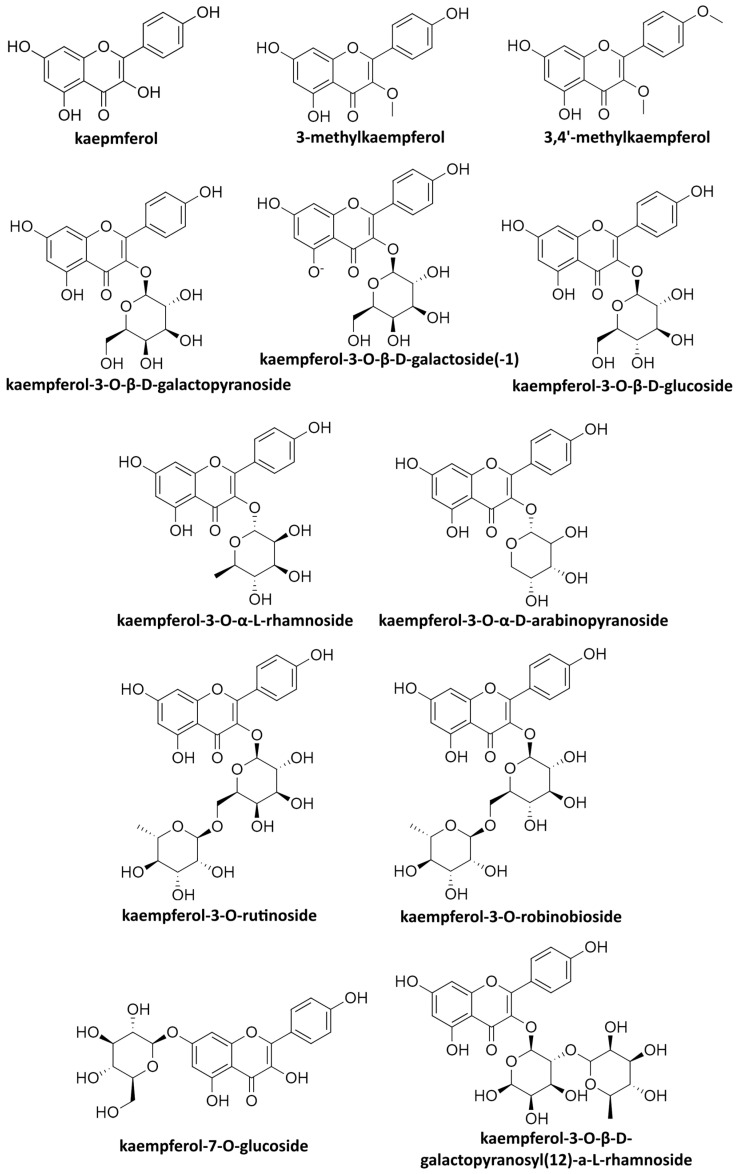
Chemical structures of kaempferol and common derivatives mentioned in the text.

As mentioned, kaempferol and its derivatives show pronounced antibacterial effects along with a considerable antifungal and antiparasitic activity [3]. Coupled with the fact that flavonoids in general have been shown to exert a promising activity against viruses [60,61], a closer view on the antiviral activity of kaempferol is warranted. In this review, we will perform a thorough analysis of the available research on the antiviral properties of kaempferol and its derivatives (Figure 1) either in pure forms or as plant extract components. This approach aims to underline the contemporary perspective of this particular field and reveal potential avenues for future research.

## 2. Antiviral Activity against DNA Viruses

In general, it is believed that DNA viruses have been evolving for millions of years [62]. There exist 91 species of DNA viruses known, and of these, about 87% exhibit a degree of adaptation to the human host [63]. Well-known human pathogens, such as the herpes viruses, adenoviruses, poxviridae and human papillomaviruses, are included in this category. Despite vaccines being available, especially after the 1930s [64] against a number of DNA viruses, they nonetheless represent a significant health hazard. Research on the antiviral properties of kaempferol and its compounds is centred on African swine fever virus, hepatitis B virus, the alphaherpsevirinae, and pseudorabies virus (Table 1).

### 2.1. Antiviral Activity against Hepatitis B Virus (HBV)

HBV infection is a major healthcare concern worldwide [72,73,74] and is responsible for a considerable number of deaths [72,73,74]. The virus is transmitted through blood and other bodily fluids [73,74] and causes liver disfunction [75] and potentially chronic hepatitis [72,73,75]. There are several regimens available for treatment purposes [72,73] which significantly reduce the risk of occurrence of complications [72,73,74,75] though there is no evidence to suggest that treatment for the acute stage is effective. At any rate, tenofovir, entecavir, and pegylated interferon alfa-2a are the main agents applied against this pathogen [75]. As far as prevention is concerned, the vaccine currently in use has been successful in limiting the prevalence of the virus [73,74,75,76], and its effectiveness is particularly important for the main risk groups, young children and pregnant women [73,76].

Kaempferol has exhibited antiviral action against enveloped viruses, including hepatitis B [77]. Moreover, in an in vitro setting, Parvez et al. [66] noticed that anti-hepatitis B effects were displayed by the extract of the cold-adapted sea buckthorn *Hippophae rhamnoides*, which contains kaempferol. Kaempferol exerted these effects by inhibiting HBsAg and HBeAg synthesis (HBe is a marker for cccDNA replication and, by extension, the infectivity of the host). An in vitro and in silico molecular docking analysis proved the ability of quercetin, kaempferol and lamivudine to form stable complexes with HBV-polymerase binding-pocket amino acids and thus, the potential therapeutic potential of sea buckthorn was attributed to its extracts, quercetin, isorhamnetin and kaempferol [66]. The findings of Li et al. [65], which used the extract of *Geranium carolinianum* L., further support the anti-hepatitis B effects of kaempferol by displaying a reduction in intracellular viral DNA proportional to the administered dose.

### 2.2. Antiviral Activity against Alphaherpesvirinae

Herpes is among the oldest pathogens recorded, being known since the time of Ancient Greece [78,79]. The two serotypes HHV-1 and HHV-2 [79], alongside Varicella–Zoster virus, make up the Alphaherpesvirinae subfamily [79]. The virus is widely distributed [80,81], and it is responsible for a multitude of pathologies of varying severity [78,80,82,83,84]. The diseases most commonly associated with herpes simplex are facial and genital lesions [80,81] as well as a recurring and potentially eyesight-threatening keratitis [85], while the psychological implications of the aforementioned lesions constitute another important aspect that must be considered during treatment [80,81]. Recent advances like the use of PCR and the administration of acyclovir have enabled us to detect the infection reliably and help the host fend it off respectively [84]. Also, new studies have shown progress in the development of vaccines with the application of nanoparticles [86].

In the case of varicella-zoster, the primary infection causes a condition commonly known as chickenpox, with the virus establishing latency afterwards in the peripheral ganglia [87,88,89], with potential reactivation years later [87,88]. The ensuing condition, i.e., herpes zoster, is commonly accompanied by potent neuralgia and may lead to CNS complications [87]. PCR and acyclovir represent solid means of detecting and addressing the virus [88,90], and available vaccines against varicella-zoster virus provide protection from both the initial infection and its reactivation [88].

Based on the results of previous research, as summarised by Yang et al. [77], it was demonstrated that kaempferol has potent action against enveloped viruses, such as herpes. Two kaempferol derivatives, kaempferol 3-O-rutinoside and kaempferol 3-O-robinobioside, obtained from the extract of *Ficus benjamina*, were shown to have inhibiting action against both HHV-1 and HHV-2 but were not effective against varicella-zoster [67]. This effectiveness is corroborated by the findings of Behbahani et al. [68], which also display the potency of kaempferol and kaempferol-7-O-glucoside isolated from *Securigera securidaca* against HHV-1 due to the ability of the tested fractions to inhibit HSV-1 multiplication at a high rate. Interestingly, a flavonoid fraction from leaves of *Ocotea notate*, with kaempferol being one of its major compounds, exhibited notably antiherpetic action, particularly against HHV-2 by inhibiting the viral replication cycle at multiple levels [91].

In the case of varicella-zoster, Park et al. [69] looked into the potency of kaempferol compared to that of acyclovir; they found out that while the former could not inhibit the VZV entry into host cell and the activity of the VZV immediate-early promoter, it could block VZV DNA synthesis and VZV replication at similar time points. Since no blocking of early, late or immediate-early proteins synthesis was observed, the mechanism behind this is possibly attributed to inhibition of the viral DNA polymerase and/or cellular factors needed for the viral DNA replication.

### 2.3. Antiviral Activity against African Swine Fever Virus

African Swine Fever (ASF), a fatal disease in many cases, is a very contagious haemorrhagic disease [92] caused by the African Swine Fever Virus (ASFV) which originates in Africa but, due to the increased demands for swine consumption, it has found its way to other areas of the world [93]. Even though this virus poses no direct threat to human life, it is a very problematic factor for the pig industry, which in turn could have repercussions as far as the availability of pork meat is concerned as well as at an economic level [92]. Seeing as how vaccine development has yet to yield any noteworthy results [94], methods of treatment could be very useful in addressing the issue alongside the implementation of strict sanitary protocols.

ASFV targets monocytes and macrophages [93]. The virus enters the cells mainly via endocytosis, mediated by receptors, and via micropinocytosis [70]. Kaempferol’s potency in dealing with this pathogen stems from its ability to inhibit the endocytosis, thereby preventing the release of virions to the cell [70]. This results in the suppression of viral infection by an impressive amount of more than 90% [70]. The mechanism behind this has not been verified, but it may be related to the induction of autophagy in different cell lines due to the upregulation of p-AMP-activated protein kinase [70].

### 2.4. Antiviral Activity against Pseudorabies Virus

This virus, also known as Suid alphaherspesvirus 1, is the causative agent of Aujeszky’s disease, which is associated with significant financial losses in the pig industry [71,95,96,97]. Even though currently pigs are its only known reservoir, it exhibits notable recombination abilities [97] and can thus affect several other mammals [96,97] with a few cases of even human infection, in the form of endophthalmitis [98] and encephalitis [99], having been recorded. DIVA (differentiating infected from vaccinated animals) vaccines have been used to address this pathogen with impressive results [100]. However, the wild swine remain a possible reservoir, and research has been inconclusive as to the risk they pose in this regard [96]. It should also be noted that on an experimental level, this virus has been used alongside other neurotropic viruses to study brain organization, as a tracer of neural pathways, and the role that virally encoded proteins play in viral invasiveness and virulence [95,101].

Kaempferol could be potentially used to treat PRV infection as it has been shown to inhibit the virus’ replication in a dose-dependent manner when tested in vitro [71]. Specifically, at a concentration of 52.40 µmol/L, it decreased cell death caused by PRV by 90%, having an IC_50_ of 25.57 µmol/L [71]. Kaempferol also exhibited inhibitory action on the stage of viral penetration though at a lesser extent, reducing the viral loads by 4-fold when compared with the 30-fold reduction in the case of replication [71].

## 3. Antiviral Activity against RNA Viruses

The RNA viruses group comprises some of the most dangerous viral pathogens, such as dengue fever virus, poliomyelitis virus, and human immunodeficiency virus (HIV). The low fidelity of their polymerases and reverse transcriptase ensures a high mutagenicity, and thus, they exhibit much greater diversification compared to DNA viruses; to date, 158 species of RNA viruses are known to exist [102] of which a handful is pathogenic to humans [63].

While successful vaccines against some of these viruses do exist, it still remains important to explore alternative treatment options, given the existence of immunocompromised individuals and the potential for mutations. There is relevant literature data to support the antiviral properties of kaempferol and its compounds against RNA viruses (Table 2).

### 3.1. Antiviral Activity against Severe Acute Respiratory Syndrome-Related Coronaviruses

Severe acute respiratory syndrome coronaviruses (SARS-CoV) are extremely contagious [118,119,120,121], and SARS-CoV-2 was the cause for the most recent pandemic, which started on 2019 and escalated into a global crisis [122]. It gains entry to the cells of the respiratory system through the binding of protein S to the angiotensin-converting enzyme [118,119,123], but apart from its main target, it can spread to several other anatomical sites [124], such as the brain [125]. Consequently, the virus manifests in several different ways; from acute respiratory distress syndrome [119,123,124,125] to gastrointestinal symptoms and kidney involvement [123,124] as well as hepatic involvement, myocardial injury, and severe coagulopathy, and it even affects the CNS [123,125,126]. Having the largest genome among all RNA viruses, SARS-CoV-2 is equipped with the means of thriving in a wide range of environmental conditions and hosts [122]. Due to its high recombination potential [125], new ways of addressing the infection must be sought after, especially given that treatment options are still limited [122]. The vaccines which were used en masse may have been mostly safe and quite efficient [127]; however, there have been reported cases of vaccine-associated pathologies [128,129,130,131].

A number of studies have assessed the efficacy of kaempferol in combating SARS-CoV-2 [132,133]. In 2014, Schwarz et al. [103] determined that numerous kaempferol glycosides were effective in blocking the 3a channels of coronaviruses in concentrations as low as 20 μΜ. This mechanism is effective against the original SARS-COV-1 outbreak of 2002–2004 [43]. Both kaempferol and isokaempferide were evaluated for their antiviral potential against COVID-19 using a computational approach and were found to be acceptable as potential antiviral agents [134]; both compounds were extracted from *Artemisia annua*. A number of kaempferol compounds extracted from *Salvadora persica* exhibited promising molecular docking parameters on the N3 site in the SARS-CoV-2 main protease (Mpro) [135]. Equally promising molecular docking parameters of kaempferol extracted from *Mollungo Nudicaulis* were confirmed by Kanmani [136].

### 3.2. Antiviral Activity against Respiratory Syncytial Virus (RSV)

This pathogen is the main causative agent of pneumonia and bronchitis [137] as well as bronchiolitis [138,139], particularly in children and the elderly [139], and it can lead to long-term pulmonary complications [139,140]. Vaccine development was arduous with the first unsuccessful attempt being made more than fifty years ago [139], although the current research results seem promising, especially since reinfection has been well documented and treatment remains for the most part supportive to this day [138,139,141].

Both kaempferol-3-O-β-D-glucopyranosyl (12)-α-L-rhamnoside and kaempferol-3-O-α-L-rhamnoside, derived from *Eucalyptus citriodora* leaves, proved effective against this virus but with a slightly lower selectivity compared to ribavirin [104]. In addition, tested kaempferol derived from tea proved effective against RSV, being able to inhibit viral cytopathic effects by 50% at a concentration of 4.84 μg/mL [77].

### 3.3. Antiviral Activity against Influenza Virus

The influenza virus is a very contagious pathogen whose main target is the respiratory system [142]. One of this virus’ most notable traits is its antigenic variability, which is most prevalent in Type A, resulting in several pandemics [143] caused by the exhibited antigenic shift [144], while its antigenic drift forces us to update the vaccines against it on an annual basis [145]. A variety of antiviral drugs are in use in influenza infections [146]; however, not only do these drugs become less effective if they are not administered shortly after the onset of the infection [143], but the virus has developed resistance to several of them [143,146]. As such, the need for additional combative substances is ever present.

Kaempferol and a host of other flavonoids are effective viral neuraminidase inhibitors [147]. The subsequent research of Jeong et al. [105] on the extract from *Rhodiola rosea roots* determined that kaempferol was an effective antiviral agent with an EC50 of 18.50–32.50 μM depending on the viral strain. This neuraminidase inhibition was also noted by Yang et al. [77]. Interference with the hemagglutinin domains has also been noted [77].

Two kaempferol compounds from the extract of *Eupatorium perfoliatum* L. were found to be effective against influenza A, although their precise contribution to the total antiviral potential of the extract was unspecified [107]. The action mechanism of the extract consisted in obstructing viral attachment and entry into the cells. The earlier research of Park et al. [148] had indicated the antiviral potential of the extract of *Aronia melanocarpa* against influenza; while the extract contains kaempferol, its exact contribution to the antiviral properties of the extract is not known. Finally, the extensive research of Kai et al. [106] on kaempferol from Brazilian propolis indicated its effectiveness in supressing viral growth in the respiratory tract with an EC_50_ of 21.70–38.20 μM depending on the viral strain by exhibiting its potency in preventing mean body weight reduction and increasing the survival rate of infected mice overall; this effectiveness was not affected by whether the strain had developed drug resistance or not.

### 3.4. Antiviral Activity against Human Immunodeficiency Virus (HIV)

It is estimated that around 39 million people were living with HIV at the end of 2022 around the world [149]. The most detrimental effect of HIV is the eventual enfeeblement of the patient’s immune system, due to the mass depletion of CD4^+^ helper cells, which is a state commonly known as AIDS (Auto Immune Deficiency Syndrome) [150,151]. A problematic aspect of this pathogen is that being a “retrovirus”, HIV uses reverse transcriptase to copy its RNA genome into DNA, showing great variability allowing for viral strains to elude the immune response of the host and making it seemingly impossible to vaccinate against [150,152]. Lifelong treatment schemes called ART (Antiretroviral Therapy) [153,154] and prophylactic schemes are now available [154,155]; therefore, our ability to mitigate the consequences of infection with this pathogen have vastly improved [154], especially if the treatment begins during the early stages [153]. On the other hand, reduced adherence to therapy, for a variety of factors [156,157,158], reduces its effectiveness [159]. For this and the other reasons outlined above, it has proven impossible to completely cure HIV carriers with a few exceptions [160].

There is some evidence to suggest that kaempferol could be used effectively as an anti-HIV agent. Behbahani et al. [108] extracted kaempferol and kaempferol-7-O-glucoside from *Securigera securidaca* and determined that it potently inhibited the activity of the viral reverse transcriptase with the kaempferol glycoside being more potent in that regard. A more miscellaneous action of kaempferol against HIV is mentioned by Badshah et al. [43] and consists of its ability to inhibit the Vpu (Viral protein u)-mediated current of HIV-1 (an ion channel that is involved in virus release when activated) by a small amount of 10% at a concentration of 20 μM in a manner similar to that of genistein [113].

### 3.5. Antiviral Activity against Dengue Fever Virus (DFV)

This pathogen has a zoonotic transmission [161], and infections caused by it vary in severity; they can be subclinical and self-limiting or cause notable fever, which can be of haemorrhagic nature in the more severe cases, potentially leading to dengue shock syndrome [161,162]. There is no dedicated antiviral treatment; however, several vaccines have been developed for children and adolescents living in endemic areas as well as subjects previously confirmed with dengue fever [163,164,165,166]. Therefore, it is of great importance to conduct research with the purpose of identifying new substances which can be used against the virus.

Kaempferol 3-O-β-rutinoside seems to be effective in inhibiting dengue virus which was attributed to its ability to interfere with molecular docking [103]. Indicatively, even at a concentration of 10 μΜ, it achieved an inhibition of 55.60% and an inhibition of 77.7% at a concentration of 100 μΜ [110]. Conversely, the earlier research of Care at al. [109] had determined that not only kaempferol was ineffective against this particular virus, but it even increased infectivity in a particular cell line. This highlights a significant aspect, namely that of different interactions between kaempferol and presumably other flavonoids with the different molecular replication strategies of different viruses.

### 3.6. Antiviral Activity against Japanese Encephalitis Virus (JEV)

Japanese encephalitis is a zoonotic disease that is endemic in regions of the Eastern hemisphere [167] but is nevertheless of global concern due to its potential to spread in new regions; indicatively, its genetic material was recently found in mosquitos in northern Italy [168]. The causative agent is a neurotrophic virus of the flavivirus genus by the same name [169] which has five circulating genotypes [167] and leads to death of the infected individual in approximately one-third of cases, with half of the surviving patients suffering from neuronal complications long after the infection is over [170]. Initially, a mouse brain-derived inactivated vaccine was available, but it has been replaced by three cell culture-derived vaccines [171].

In general, flavonoids seem to be promising antiviral agents against JEV [70,111,172]. Kaempferol in particular was found to be able to bind with the frame-shift site RNA (fsRNA) in the JEV serogroup and thereby interfere with protein expression and consequently viral replication [111]. The antiviral action of kaempferol against this virus was also corroborated by Care et al. [109], even though higher concentrations of kaempferol were required, and this was above the toxicity limit for one of the two cell lines used.

### 3.7. Antiviral Activity against Enterovirus 71 (EV71)

Enterovirus 71 is the main causative agent of the so-called “hand, foot, and mouth disease” [173,174,175] alongside coxsackieviruses [174], which is a condition that affects mostly children [173,175,176], having neurological manifestations [173,175,176], and can lead to complications both neurologic [175] and systemic [173,175], like meningoencephalitis and pulmonary pathologies, respectively. Currently, there are formalin-inactivated (FI) EV71 vaccines which have undergone evaluation in human clinical trials in China, Taiwan, and Singapore and have been deemed safe and effective in eliciting neutralizing antibody responses against the virus [174].

Kaempferol can be potentially useful against enterovirus 71 infections because it hinders both its replication and its translation through its interaction with EV71 internal ribosome entry site (IRES) [112]. Indicatively, PCBP 1/2 and the viral polymerase precursor 3CD with poliovirus IRES stem-loop I RNA contribute significantly to the antiviral activity by forming a ternary complex required for the synthesis of negative-strand RNA while PCBP2 interacts with enterovirus IRES stem-loop IV RNA thus inhibiting IRES-mediated translation [112]. Kaempferol’s action against enterovirus 71 is made possible due to the contribution of cellular factors associated with the 50-untranslated region (50-UTR) of the EV71 genome [112].

### 3.8. Antiviral Activity against Hepatitis A Virus (HAV)

Hepatitis A is the most frequent type of viral hepatitis and is showing an increasing incidence especially in low and middle demographic index regions [177]. The faecal–oral transmission shows it is oftentimes linked to poor sanitary conditions [178]. The infection can have many clinical forms, from being short-lived and self-limiting, without displaying any symptoms [179] or displaying abdominal pain, hepatitis, hyperbilirubinemia, and jaundice [178] or only jaundice, to being seldom fatal [179]. Intramuscular anti-A gamma globulin is used for passive immune prophylactic purposes [179], but the most efficient preventive method is the available vaccine [178,179,180].

As mentioned in the research of Ohemu et al. [181], a variety of plant flavonoids, presumably including kaempferol, showed an antiviral effect against HAV. However, Orabi and Orabi [114] tested kaempferol-3-O-α-D-arabinopyranoside and kaempferol-3-O-β-D-galactopyranoside isolated from the extract of *Ficus virens*; their antiviral activity against HAV was found to be practically non-existent. Both compounds were tested up to their maximum non-toxic concentration on the tested cell line.

### 3.9. Antiviral Activity against Poliovirus

Poliovirus is a virus with three subtypes (1, 2 and 3), which is responsible for the condition known as poliomyelitis [182]. This enterovirus is highly contagious and can have either a paralytic form (most often spinal) or a bulbar form [182]. In addition to the dangerous nature of the acute phase, this virus remains a problem for the host’s health for a long time after the initial infection due to post-polio syndrome, which is a neurological disorder that significantly impairs his daily life [182,183,184], requiring long-term medical assistance to handle [183]. The vaccines of Salk and Sabin were a major step toward limiting the impact of this pathogen [182,184]. Apart from being effective, they were also relatively safe [185,186] even though some cases of vaccine-derived polio have been recorded [185].

Based on the research of Robin et al. [115], 3-methylkaempferol (also known as isokaempferide) and 3,4-dimethylkaempferol, two compounds derived from the leaves of *Psiadia dentata*, have been found to be very effective in preventing viral replication of the second subtype by inhibiting RNA synthesis of the plus-strand if administered shortly after the infection.

### 3.10. Antiviral Activity against Chikungunya Virus (CHIKV)

This pathogen has a zoonotic transmission and causes fever, often accompanied by a maculopapular rash, culminating into polyarthralgia of potentially chronic nature [187,188,189], which can even cause bone erosion [189], while it has also been associated with Guillain–Barré syndrome [190]. Although the disease is usually not life-threatening, some patients develop chronic joint pain possibly due to the migration of infected monocytes to the synovial tissues, perpetuating the inflammatory process [188,189]. Given that all attempts at creating a vaccine have not been successful thus far [189] and that there is no specific treatment available [187,188,190], with broad-spectrum antivirals like ribavirin currently comprising the most effective therapeutic scheme available [189,191], research has focused on finding natural compounds with action against this virus [190].

Kaempferol with the oxidation and cyclization of 1,3,5-trimethoxybenzene was found to inhibit replication after the entry of the virus at a concentration > 400 µM [191]. As such, kaempferol could be considered a possible CHIKV inhibitor for human use. On the other hand, based on the research of Lani et al. [116], kaempferol is not a good candidate as an antiviral agent against CHIKV, because even though it exhibits a degree of inhibition of post-viral replication [191], it does so at a very high concentration of 400 µM [116].

### 3.11. Antiviral Activity against Feline Calicivirus (FCV)

This pathogen is among the most common ones found in cats [192,193] causing sporadic outbreaks in that population [194]. It has a highly mutagenic nature with its great genomic plasticity making the diagnosis, treatment, and prevention quite challenging. Infecting the upper respiratory tract [193,194], it is commonly accompanied by oral ulcers and high fever [195], and it can even be fatal [192]. Treatment has only a supportive role [195] and, despite the effectiveness of the vaccine [193,195], the virus’ variability means that there are strains against which the protection granted is not as potent [192]. Although this particular virus is not pathogenic to humans, it belongs to the *Caliciviridae* family, which comprises notable human pathogens [196]. Therefore, the development of antiviral strategies against this virus may offer new insights against human pathogens.

The research of Seo et al. [117] shows that kaempferol was the most potent antiviral agent amongst a series of tested flavonoids; compared to the standard antiviral agent ribavirin, it proved by far less effective at a concentration of 50 μM and somewhat less effective at 100 μM. The inhibition by kaempferol was remarkably high at concentrations of 200 μM and 300 μM [117]. An important component of kaempferol’s effectiveness seems to be its antioxidant activity, which surpasses even that of ribavirin [110].

### 3.12. Antiviral Activity against Murine Norovirus (MNV)

This recently discovered pathogen affects mice, causing gastroenteritis [197]. There also is a strain which can affect humans, called human norovirus, which is the main causative agent of viral gastroenteritis worldwide [198,199]. Since the mechanisms of the human norovirus have yet to be fully elucidated, despite recent advances [198,199], the study of the murine norovirus could yield results that may culminate into a breakthrough, especially since the latter is unique among all the strains in its ability to replicate in a cell culture [197].

While belonging in the same family as FCV, murine norovirus was not inhibited by kaempferol at either 50 μM or 100 μM despite other flavonoids such as daidzein and quercetin being effective against it [117].

## 4. Effectiveness of Kaempferol Antiviral Activity

Another aspect we should consider is the relative effectiveness of kaempferol and its derivatives compared to existing antiviral drugs (Table 3). In a large number of cases, the compounds tested are of comparable effectiveness to the drugs used, which is important when considering the potential adverse effects of the antiviral drugs. Thus, it might be possible to partially supplant or supplement these drugs, with kaempferol either alone or in conjunction with other natural antiviral compounds, so as to reduce the dose and/or the length of administration of some antiviral drugs, especially in the case where serious side effects are manifested.

## 5. General Antiviral Activity and Natural Kaempferol Sources with Antiviral Effects

**Figure 2:**
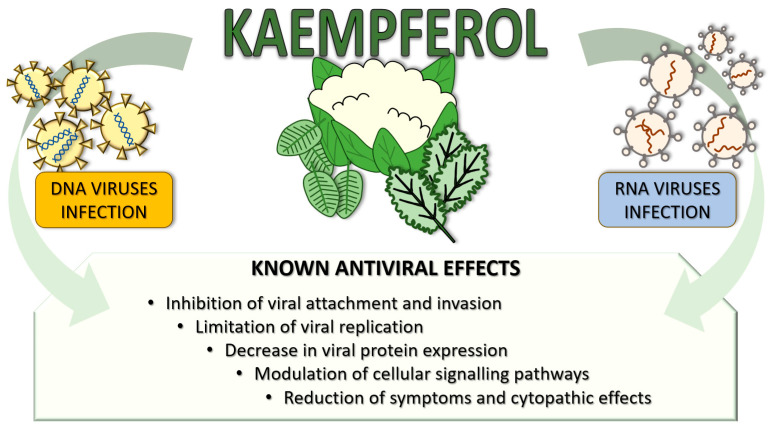
Summary of kaempferol antiviral effects on DNA and RNA viral infections.

From all the aforementioned research results on the antiviral effects of kaempferol, it must become apparent that kaempferol and its derivatives are potent as antiviral agents against most of the viruses presented (Figure 2). In each experiment against DNA viruses, every compound tested was effective at reasonably low concentrations, which were below the toxicity limit for the cell lines tested by the same researchers. Regarding kaempferol toxicity, there is currently no consensus as to its safety profile in vivo [7]; in vitro studies [200,201,202] have found it to be genotoxic, but this effect was not replicated in vivo [203].

**Figure 3:**
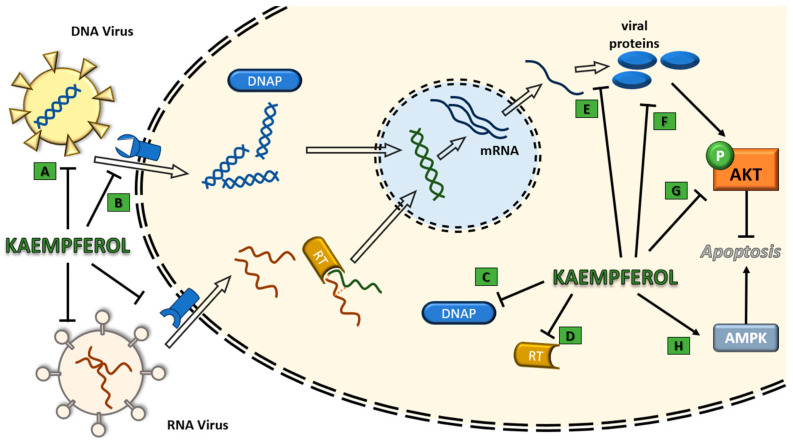
Illustration of the action mechanisms of kaempferol and its derivatives on viral entry, replication and effects. A. Direct effect on viral molecules; B. Inhibition of viral attachment; C. Inhibition of polymerase activity; D. Inhibition of reverse transcription; E. Inhibition of viral proteins transcription; F. Inhibition of viral proteins activity; G. Down-regulation of the PI3K-Akt pathway; H. Up-regulation of AMPK signalling. DNA = deoxyribonucleic acid, RNA = ribonucleic acid, DNAP = deoxyribonucleic acid polymerase, RT = reverse transcriptase, AKT = protein kinase B, AMPK = adenosine monophosphate-activated protein kinase.

In the case of RNA viruses, the results are again encouraging, with kaempferol and its derivatives being able to either inhibit viral replication altogether or mitigate the cytopathic effects and inhibit the entry of viruses into the cells. Notable exceptions include the experiment of Care et al. [109] against dengue, the experiments against HAV [114] and the experiments of Sauter et al. [113] against HIV; in all cases, the compounds used were ineffective. In the case of dengue virus, it is important to explore the potential of other kaempferol compounds, given that it represents a notable public health hazard in certain countries, and vaccination was not yet successful [204]. It is worth noting that kaempferol seems to exhibit some sort of cell specificity [109]. In this study, kaempferol was even found to have a pro-viral effect, which was most probably associated with the presence of a functional IRES sequence in Dengue virus [205]. In the case of HIV, kaempferol elicited only a minor inhibition of the Vpu channel [113]. Finally, in the case of kaempferol compounds against HAV, the cause for the lack of any noticeable antiviral activity appears to be related to structural and molecular dynamics reasons [114]. The ability of kaempferol and its derivatives to inhibit viral replication was identified and described for both DNA and RNA viruses; an illustration of the reported effects is shown in Figure 3.

Kaempferol is abundant in nature, being found in many plants (e.g., [206,207,208,209,210,211,212,213,214,215,216,217]; for a comprehensive review, see the paper of Periferakis et al. [3]). It is synthesised via the shikimic acid pathway [218] as all flavonoids; this biosynthetic pathway may be metabolically engineered so that it can yield the desired flavonol amounts in the near future [219]. Indeed, the microbial synthesis of kaempferol, as an alternative to its purification from plants, has already been proposed by Duan et al. [220]. Regarding its absorption in the human body, kaempferol is usually absorbed as a glycoside, although the attached sugars impact its bioavailability [221]. The specific pharmacokinetics of kaempferol have been explored by a number of researchers [222,223,224,225,226,227,228,229,230].

A prominent detail readily apparent based on the data presented in this review is the importance of plants used in traditional and folk medicine systems since ancient times. The mentioned plants belong to one or several such medical traditions (Table 4), and most importantly, the majority already demonstrated antimicrobial/antiviral properties with the exception of *Securigera securidaca*. This highlights the fact that a persistent and detailed study of traditional remedies can yield very useful insights into bioactive compounds for a number of different pathologies.

The possibility of virus–host genetic recombination coupled with the viral adaptability may make it plausible that a number of viruses may yet circulate with the human population unnoticed until specific and characteristic symptoms are manifested [102]. Alongside the existing viruses against which no effective vaccines are available, the need for an intensification of research on the antiviral properties of natural compounds is evident. Kaempferol appears to be a strong candidate due to its effectiveness, variety of action mechanisms, and general availability. Moreover, in an effort to enhance tissue availability, selectivity and effectiveness, kaempferol and associated compounds could be combined with nanoparticles, which have promising medical applications [265,266,267]. Another interesting future research avenue would be to combine kaempferol with other phytochemicals with known antiviral properties such as catechins [268,269,270], quercetin [271,272,273], plant alkaloids [55,274,275,276], and other natural compounds [277,278,279].

A variety of zoonotic viruses have the potential to emerge and infect human populations; therefore, the surveillance of infections and nonhuman reservoirs is recommended [280]. Further research on the antiviral properties of kaempferol on viruses from these reservoirs could be beneficial in establishing its effectiveness and potential use alongside other antiviral drugs.

## Figures and Tables

**Table 1:** In vitro studies on the potential antiviral actions and effects of kaempferol and its derivatives against DNA viruses.

Family	Genus	Extract from	Compound Tested	Toxicity Limit	Concentration (Type of Effect)	Mechanism	Year of Research	Reference
Hepadnaviridae	Hepatitis B virus (HBV)	Extract of *Geranium carolianum* L.	kaempferol	160.79 μg/mL	47.54 mg/kg (ED_50_)	Decrease in HBsAg and HBeAg and of viral DNA synthesis	2008	[65]
		Leaf ethanol extract of *Hippophae rhamnoides*	kaempferol	32.58 μg/mL (CC_50_ for SB-Chl) 150 μg/mL (SB-Eac, SB-But and SB-Aqu)	10 μg/mL (stable concentration used in the experiment)	Inhibition of HBsAg and HBeAg expression	2022	[66]
Orthoherpesviridae (Alphaherpesvirinae)	Human herpesvirus 1virus 1 (HHV-1)	Extract from *Ficus benjamina*	kaempferol 3-O-rutinoside	300 ± 2.79 μmol/L	3.00 ± 0.97 μmol/L (EC_50_)	Unknown	2012	[67]
			kaempferol 3-O-robinobioside	600 ± 10.45 μmol/L	0.90 ± 0.23 μmol/L (EC_50_)			
		Extracts of dried and powdered *Securigera securidaca* seeds	kaempferol	60.0 ± 5.0 μg/mL (CC_50_)	0.20 ± 0.12 μg/mL (EC_50_)	Inhibition of viral attachment and entry into cells; inhibition of viral polymerase	2014	[68]
			kaempferol-7-O-glycoside	250 ± 1.7 μg/mL (CC_50_)	0.20 ± 0.01 μg/mL (EC_50_)			
	Human herpesvirus 2 (HHV-2)	Extract from *Ficus benjamina*	kaempferol 3-O-rutinoside	Unknown	n/a	n/a	2012	[67]
			kaempferol 3-O-robinobioside	Unknown				
	Varicella-Zoster Virus (VZV)	Pure compound	kaempferol	No cytotoxicity detected	6.36 ± 0.73 μg/m (IC_50_)	Blockade of viral DNA synthesis and viral replication	2022	[69]
Asfarviridae	African Swine Fever Virus (ASFV)	Pure compound	kaempferol	93.10 μg/mL (CC_50_)	2.20 μg/mL (IC_50_)	Unknown	2021	[70]
Orthoherpesviridae	Pseudorabies Virus (PRV)	Pure compound	kaempferol	254.97 ± 1.86 μmol/L (CC_50_)	25.57 ± 0.74 μmol/L (IC_50_)	Regulation of the MAPK and NF-κB pathways	2021	[71]

**Table 2:** In vitro studies on the potential antiviral actions and effects of kaempferol and its derivatives against RNA viruses.

Family	Genus	Extract From	Compound Tested	Toxicity Limit	Concentration (Type)	Mechanism	Year of Research	Reference
Coronaviridae	SARS coronavirus (SARS-COV-2)	Pure compounds	Numerous kaempferol glycosides	Not calculated	20 μΜ (minimum effective concentration)	Inhibition of the 3a membrane channel	2014	[103]
Pneumoviridae	Respiratory syncytial virus (RSV)	Extract from *Eucalyptus citriodora*	Kaempferol-3-O-β-D-glucopyranosyl (12)-α-L-rhamnoside	137.60 μg/mL (CC_50_)	57.30 μg/mL (IC_50_)	Reduction in virus multiplication	2014	[104]
			Kaempferol-3-O-α-L-rhamnoside	258.1 μg/mL (CC_50_)	56.90 μg/mL (IC_50_)			
		Extract from *Sophora japonica* flowers	Kaempferol	143.79 μg/mL (TC_50_)	4.84 μg/mL (IC_50_)	Reduction in viral cytopathic effects	2014	[77]
Orthomyxoviridae	Influenza virus	Extract from *Rhodiola rosea* roots	Kaempferol	>300 μΜ	18.50–30.20 μM (EC_50_ depending on viral strain)	Inhibition of neuraminidase	2009	[105]
		Extract from Brazilian propolis	Kaempferol	>100 μg/mL	21.70–38.20 μM (depending on viral strain)	Limitation of infection symptoms	2014	[106]
		Extract from *Eupatorium perfoliatum* L.	Kaempferol-3-O-β-D-galactoside (trifolin)	Not calculated for individual compounds	Various effective concentrations	Prevention of viral attachment and entry into the cells	2016	[107]
			Kaempferol-3-O-β-D-glucoside (astragalin)					
Retroviridae	Human immunodeficiency virus (HIV)	Extract from *Securigera securidaca*	Kaempferol	320 μg/mL	50 μg/mL (IC_50_)	Inhibition of reverse transcriptase	2014	[108]
			Kaempferol-7-O-glycoside	2500 μg/mL	32 μg/mL (IC_50_)			
Flaviviridae	Dengue fever virus (DFV)	Pure compound	Kaempferol	228.50 Μμ (HEK293Τ/17 cells); 139.70 Μμ (BHK-21 cells)	None (not effective at tested concentration)	-	2020	[109]
		Extract from *Azadirachta indica*	Kaempferol-3-O-rutinoside	Not significant	10 μΜ (minimum tested concentration)	Inhibition of viral protease	2021	[110]
	Japanese encephalitis virus	Pure compound	Kaempferol	230 μΜ	12.6–21.5 μM (depending on experimental conditions)	Inhibition of viral protein expression	2012	[111]
		Pure compound	Kaempferol	228.50 Μμ (HEK293Τ/17 cells); 139.70 Μμ (BHK-21 cells)	66.33 μM (EC_50_)	Probably inhibition of cap-dependent translation	2020	[109]
Picornaviridae	Enterovirus 71	Pure compound	Kaempferol	50 μΜ<	>35 μM (standard concentration used)	Inhibition of translation and replication	2011	[112]
	Hepatitis A virus (HAV)	Pure compound	Kaempferol	Not determined	None (not effective at tested concentrations)	-	2014	[113]
		Extract from *Ficus virens*	Kaempferol-3-O-α-D-arabinopyranoside	329.9 ± 5.3 μg/mL	None (not effective at tested concentrations)	-	2016	[114]
			Kaempferol-3-O-β-D-galactopyranoside	313.3 ± 1.19 μg/mL	None (not effective at tested concentrations)			
	Poliovirus	Extract from *Psiadia dentata*	3-Methylkaempferol	107 μΜ	Various tested concentration under different settings	Inhibition of the replication	2001	[115]
			3,4′-Dimethylkaempferol	197 μΜ				
Togaviridae	Chikungunya virus (CHIKV)	Pure compound	Kaempferol	>1000 μg/mL (CC_50_ Vero cells); 537.30 μg/mL (CC_50_ BHK-21 cells)	400 μΜ (concentration necessary for a degree of inhibition)	Inhibition of post-entry replication	2015	[116]
Caliciviridae	Feline calicivirus (FCV)	Pure compound	Kaempferol	>300 μΜ	50 μΜ (minimum effective concentration)	Unknown	2016	[117]
	Murine norovirus (MNV)				None (not effective at tested concentrations)	-		

**Table 3:** Effectives of kaempferol and its derivatives relative to common antiviral drugs.

Virus	Compound Tested	Compound Effectiveness (Concentration)	Reference Drug	Drug Effectiveness (Concentration)	Reference
Hepatitis B virus	Kaempferol	10 μg/mL (62.3% viral inhibition of HBsAg synthesis)	Lamivudine	2 μΜ (87.4% viral inhibiton of HBsAg synthesis)	[66]
Human herpesvirus 1	Kaempferol	0.20 ± 0.01 μg/mL (EC_50_)	Acyclovir	0.10 ± 0.01 μg/mL (EC_50_)	[68]
	Kaempferol-7-O glycoside	0.10 ± 0.01 μg/mL (EC_50_)			
Varicella-zoster	Kaempferol	6.36 ± 0.73 µg/mL (IC_50_)	Acyclovir	0.54 ± 0.12 µM (IC_50_)	[69]
Pseudorabies virus	Kaempferol	25.57 μg/mL (IC_50_)	Acyclovir	54.97 μg/mL (IC_50_)	[71]
Feline calicivirus	Kaempferol	50–100 μΜ (tested concentrations)	Ribavirin	Higher effectiveness at the same concentrations	[117]
Influenza virus	Kaempferol	21.70–38.20 μg/mL (EC_50_)	Ribavirin	19.20 ± 7.5 μg/mL (EC_50_)	[106]
Respiratory syncytial virus	Kaempferol-3-O-β-D-glucopyranosyl (12)-α-L-rhamnoside	57.30 μg/mL (IC_50_)	Ribavirin	2.60 μg/mL (IC_50_)	[104]
	Kaempferol-3-O-α-L-rhamnoside	56.90 μg/mL (IC_50_)	Ribavirin	2.60 μg/mL (IC_50_)	
HIV	Kaempferol	50 μg/mL (IC_50_)	Zidovudine	1 μg/mL (IC_50_)	[108]
	Kaempferol-7-O-glycoside	32 μg/mL (IC_50_)			

**Table 4:** Traditional medical uses of plants mentioned in this review.

Plant	Medical Tradition	Traditional/Ethnobotanical Uses	References
*Atermisia annua*	Traditional Chinese medicine	Anti-hyperlipidaemic, anti-plasmodial, anti-convulsant, anti-inflammatory, antimicrobial, anti-cholesterolaemic and antiviral properties	[231,232]
*Azadirachta indica*	Traditional Chinese medicine, Ayurvedic medicine, Unani medicine	Antimicrobial and anti-inflammatory uses	[233,234,235]
*Eucalyptus citriodora*	African folk medicine (various geographical areas)	Anti-asthmatic, antifungal, general antimicrobial	[236,237,238,239]
*Eupatorium perfoliatum*	Native American folk medicine, European medical traditions	Antipyretic, antirheumatic agent and treatment of colds, anti-malarial agent and use as an antiviral agent	[240,241,242]
*Ficus benjamina*	Numerous local remedies in Asia, Africa, the Pacific islands and the Americas	Antimicrobial, antinociceptive, antipyretic and hypotensive uses; anti-dysentery remedy	[243,244,245,246]
*Ficus virens*	Traditional Indian medicine and Ayurveda	Prevention and treatment of diseases and various other reported medicinal effects	[247]
*Geranium carolinianum* L.	Traditional Chinese medicine	Antimicrobial, anti-inflammatory, and antipyretic uses	[248]
*Hippophae rhamnoides*	Local medical traditions in Russia and Asia, Austrian folk medicine	Treatment of hypertension, oedema, inflammation; tissue regeneration; treatment of burns, wounds, and ulcers	[249,250,251,252]
*Ocotea notata*	Folk medicine of South America	Treatment of chest pain, rheumatism wounds and viral infections	[253]
*Psiadia dentata*	Local African medical traditions, folk medicine of the Mascarene islands	Treatment of abdominal pains, colds, fevers, bronchitis, asthma, rheumatoid arthritis, skin infections and liver disorders	[254,255]
*Rhodiola rosea*	Traditional Chinese medicine, Viking folk medicine, various local medical traditions of Asian and European countries	Nervous system stimulation, stress and fatigue alleviation, treatment for gastrointestinal complaints, anaemia, infections, and impotence	[256,257,258]
*Salvadora persica*	Traditional Indian medicine, African folk medicine, medical tradition of Saudi Arabia	Antidote to poison, prevention of scurvy, treatment of rheumatism, anti-inflammatory use, treatment of skin conditions, purgative, treatment of gastrointestinal disorders, antimicrobial properties	[259,260]
*Securigera securidaca*	Traditional Iranian medicine, traditional Egyptian medicine, traditional Indian medicine	Anti-epileptic, anticonvulsant, and blood lipid-lowering actions	[261,262]
*Sophora japonica*	Traditional Chinese medicine, traditional Japanese medicine, traditional Korean medicine	Treatment of haemorrhoids, haematochezia, haematuria, hematemesis, haemorrhinia, uterine or intestinal haemorrhage, arteriosclerosis, headache, hypertension, dysentery, dizziness, and pyoderma	[263,264]

## Data Availability

Not applicable.
